# Nap Opportunity During the Daytime Affects Performance and Perceived Exertion in 5-m Shuttle Run Test

**DOI:** 10.3389/fphys.2019.00779

**Published:** 2019-06-20

**Authors:** Omar Boukhris, Raouf Abdessalem, Achraf Ammar, Hsen Hsouna, Khaled Trabelsi, Florian A. Engel, Billy Sperlich, David W. Hill, Hamdi Chtourou

**Affiliations:** ^1^UR15JS01: Education, Motricité, Sport et Santé (EM2S), High Institute of Sport and Physical Education of Sfax, University of Sfax, Sfax, Tunisia; ^2^Department of Movement and Training Science, Institute of Sport and Sport Science, Heidelberg University, Heidelberg, Germany; ^3^Department of Sport Science, Integrative and Experimental Training Science, University of Würzburg, Würzburg, Germany; ^4^Applied Physiology Laboratory, Department of Kinesiology, Health Promotion, and Recreation, University of North Texas, Denton, TX, United States; ^5^Institut Supérieur du Sport et de l’éducation Physique de Sfax, Université de Sfax, Sfax, Tunisie; ^6^Activité Physique, Sport et Santé, UR18JS01, Observatoire National du Sport, Tunis, Tunisie

**Keywords:** nap, sleep, sport, exercise, fatigue

## Abstract

**Purpose:**

To compare the effect of different durations of nap opportunity during the daytime on repeated high-intensity short-duration performance and rating of perceived exertion (RPE).

**Methods:**

Seventeen physically active men (age: 21.3 ± 3.4 years, height: 176.7 ± 5.9 cm, body mass: 71.8 ± 10.2 kg) performed a 5 m shuttle run test [to determine best distance (BD), total distance (TD), and fatigue index (FI)] under four conditions: a 25 min nap opportunity (N25), a 35 min nap opportunity (N35), a 45 min nap opportunity (N45), and control condition (no-nap) (N0). The sleep quality of each nap opportunity was evaluated using a scale ranging from 0 “no sleep” to 10 “uninterrupted, deep sleep throughout.” The four conditions were performed in a random order. RPE was recorded after each repetition of the 5 m shuttle run test and the mean score was calculated.

**Results:**

BD increased after N25 (+6%) and N45 (+9%) compared to N0 (*p* < 0.05) and was significantly higher after N45 compared to N35 (*p* < 0.05). Compared to N0, the three nap opportunity durations enhanced TD (*p* < 0.05) with greater enhancement after N45 compared to N25 (+8% vs. +3%) and N35 (+8% vs. +3%). For FI, no-significant differences were observed between the three nap opportunity durations and N0. The mean RPE score was significantly higher after N25 (+20%) and N0 (+19%) compared to N45 (*p* < 0.05). All participants were able to fall asleep during each nap condition with a sleep quality score of 6.9 ± 1.0, 7.0 ± 0.7, and 7.1 ± 0.8 for N25, N35, and N45.

**Conclusion:**

A nap opportunity during the daytime was beneficial for physical performance and perceived exertion with the N45 being the most effective for improving performance and reducing fatigue during the 5 m shuttle run test. The implication of the present study is that athletes might benefit from a nap opportunity of 25, 35 or 45 min before practice or before a competition.

## Introduction

Wakefulness and sleep are modulated by the internal biological clock located in the brain at the hypothalamus, and precisely, in the suprachiasmatic nuclei (Van Dongen and Dinges, 2000). The impact of the biological clock goes beyond governing sleep and wake processes (Van Dongen and Dinges, 2000). It regulates the sleep/wake system and many other functions, such as blood pressure, hormone levels, body temperature, physical performance, alertness, mood and intellectual abilities that fluctuate during the day (Davenne, 2009).

It is well known that the majority of human cognitive performance follows a circadian rhythm with peak performance occurring in the early evening and lower performance in the afternoon (the so-called “post-lunch dip”) (Schmidt et al., 2007). The post-lunch dip is a period of sleepiness that occurs between 13h00 and 16h00 due in part to a slight reduction in core body temperature, which promotes a tendency to sleep (Van Dongen and Dinges, 2000) and causes a temporary decrease in vigilance (Monk, 2005). The post lunch-dip is not completely explained by meal ingestion, but, rather reflects the 12 h harmony (i.e., 12 h sinusoids; e.g., the size of the 12 h component rhythm amplitude of core temperature) of the circadian clock (Monk, 2005).

Athletes must face the potential impact of the post-lunch dip when they compete or train in the afternoon (Winget et al., 1985). The magnitude of sleepiness caused by the post-lunch dip is increased by fatigue, stress, and/or sleep loss (Winget et al., 1985; Nédélec et al., 2015). Since a nap longer than 30 min is composed of slow-wave sleep, which helps in recovery (Waterhouse et al., 2007), athletes could implement a short sleeping period during the daytime (napping strategy) to overcome the decline in alertness and performance caused by the post-lunch dip (Ficca et al., 2010). In this context, it has been reported that subjective sleepiness decreased after a nap of 15–45 min during the post-lunch dip (i.e., around 14h00) (Hayashi et al., 1999; Horne and Reyner, 2001). Additionally, several studies suggest that a nap could reduce fatigue and improve vigor, subjective alertness, objective vigilance, and cognitive performance (Brooks and Lack, 2006;Verweij et al., 2016). Recently, Petit et al. (2018) showed that a 20 min nap has a beneficial effect on information processing (using the P300 test which measures the positive deflection at about 300 ms in response to a rare stimulus), alertness, and cognitive processing in athletes. O’Donnell et al. (2018) showed that a short nap (i.e., <20 min) opportunity enhanced the subjective estimation of performance in elite netball players (i.e., by coaches) and the jump velocity during the day of the competition. A few studies have examined the effects of napping on physical performance (Waterhouse et al., 2007; Petit et al., 2014; Blanchfield et al., 2018; Hammouda et al., 2018; Keramidas et al., 2018; Daaloul et al., 2019). In this context, Waterhouse et al. (2007) examined the effects of a 30 min post-lunch nap in partially sleep-deprived athletes and showed that a 20 m sprint performance was improved with napping. Recently, Hammouda et al. (2018) reported that a 20 min nap and a 90 min nap, taken after partial sleep deprivation, improved repeated-sprint performance and that the longer the nap duration, the greater was the improvement. Similarly, Blanchfield et al. (2018) reported that a short afternoon nap (34 ± 12 min in bed with 20 ± 10 min asleep time) improved endurance performance in runners who had obtained less than 7 h of night-time sleep. In addition, Keramidas et al. (2018) showed that a 30-min nap may lessen physical performance reduction engendered by short-term multi-stressor military training. In the same way, Daaloul et al. (2019) reported that a 30-min nap has a beneficial effect on physical performance (i.e., karate specific test, squat jump and counter movement jump). However, Petit et al. (2014) found no significant effects of a 20-min nap on peak 5 s power during the Wingate test in athletes either after normal sleep or after a 5-h phase advance sleep.

In light of the existing literature, while advising athletes to include a nap during the day seems reasonable, there seems to be little empirical evidence as to whether a nap really is beneficial for athletic performance and, if it is, what nap duration is best for recovery. The effect of a nap on repeated-sprint performance has been studied after a night of disrupted sleep, but to the best of our knowledge, no previous study has examined this effect after one night of normal sleep. In addition, previous studies have demonstrated that the length of a nap affects its effectiveness in improving short-duration high-intensity performance (Hammouda et al., 2018). Therefore, the purpose of this study was to examine the effect of different nap opportunity durations on short-duration high-intensity repetitive performance and the corresponding rating of perceived exertion (RPE). The present study examined three durations of nap opportunity during the daytime: 25 min (N25), 35 min (N35), and 45 min (N45). Based on previous studies showing an improvement (or a greater improvement) in physical performance when nap durations were 30 min or more (e.g., Keramidas et al., 2018; Blanchfield et al., 2018; Daaloul et al., 2019), we hypothesized that a greater improvement in a short-duration high-intensity performance and a greater reduction in RPE scores would be observed in the N45 condition than in the N35 condition, the N25 condition, and the control no-nap condition (N0).

## Materials and Methods

### Participants

Seventeen physically active men (age: 21.3 ± 3.4 years, height: 176.7 ± 5.9 cm, body mass: 71.8 ± 10.2 kg) voluntarily participated in the present study. After receiving a description of the protocol, including potential risks, and benefits, participants gave their written consent to participate in the study. The criteria for participants’ inclusion were: they were non-smokers, they did not have pathological sleep disorders, they did not consume alcohol, and they were regularly engaged in training (e.g., jogging) for ≈1 h per day, 3 days per week.

Additionally, to avoid any undesirable effect of sleep deprivation, participants were asked to keep usual sleep duration (≈7 h) throughout the experimental period. Although there was no objective measurement of sleep, compliance with this instruction was verified using sleep diaries (i.e., a sleep duration recorded in sleep diaries of ∼8 h for all conditions; for details see Table 1) and the Pittsburgh Sleep Quality Index (PSQI)(i.e., a total PSQI score of 2.9 ± 1.7) (Suleiman et al., 2010). The present study was conducted according to the Code of Ethics for human experimentation, the Declaration of Helsinki (World Medical Association, 2013) and the protocol was fully approved by the Research Ethics Committee of the High Institute of Sport and Physical Education of Sfax, University of Sfax, Tunisia before the commencement of the assessments.

**Table 1:** Best distance (BD), total distance (TD), fatigue index (FI), rating of perceived exertion scale (RPE) and sleep quality recorded after the no-sleep condition (N0) and the 25-min (N25), the 35-min (N35) and the 45-min (N45) of nap opportunity during the daytime.

	N0	N25	N35	N45
BD (m)	126.4 ± 13.6	134.1 ± 13.4^∗^	131.1 ± 7.8^#^	139.6 ± 15.9^∗^
TD (m)	697.1 ± 74.1	719.9 ± 65.5^∗#^	720.5 ± 52.2^∗#^	755.1 ± 63.3^∗^
FI (%)	11.7 ± 3.2	13.3 ± 6.0	10.5 ± 5.8	10.8 ± 2.4
RPE	4.6 ± 1.1^#^	4.8 ± 1.5^#^	4.6 ± 1.2	3.7 ± 1.1
Sleep quality	–	6.9 ± 1.0	7.0 ± 0.7	7.1 ± 0.8

*^∗^Significant difference compared to N0; ^#^Significant difference compared to N45.*

### Experimental Design

**FIGURE 1:**
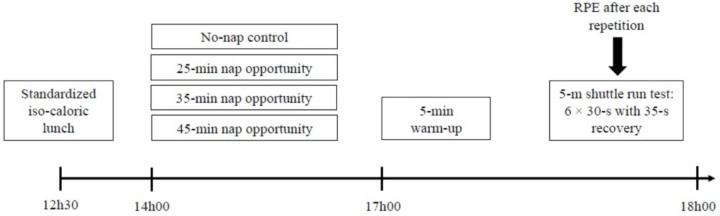
Schematic representation of the experimental protocol. RPE, Rating of perceived exertion.

After an initial familiarization session for the study protocol, participants randomly attended four test sessions (N0, N25, N35, and N45) with at least 72 h in-between. A free-online software resource^1^ was utilized to counterbalance and randomize the order of the test sessions. For each test session, participants woke up at 07h30 ± 30min and ate a standardized iso-caloric lunch at 12h30. From 12h30 onward, participants were allowed to consume only drinking water. After a rest period of 75 min, they got into bed at 13h45 in sleep rooms that were favorable to sleep (i.e., fully dark, and quiet). After 15 min to become accustomed to their new place of sleep, participants were asked to take a nap opportunity from 14h00 to (i) 14h25 for N25, (ii) 14h35 for N35, or (iii) 14h45 for N45. For the N0 and the nap opportunity conditions, participants spent the remaining time until 17h00 reading books, watching videos on television, or playing video games in a comfortable arm chair. In order to observe the participants’ activities, sleep rooms were constantly monitored using an infra-red camera connected, in real-time, to the experimenter’s computer. Although, there was no objective measurement of sleep during the nap opportunity, participants were asked about their subjective sleep quality after awakening using a scale of 0–10 points, where 0 indicated “no sleep,” 5 indicated “some sleep with some interruptions,” and 10 indicated “uninterrupted, deep sleep throughout.”All participants were able to fall asleep during each nap condition (i.e., a sleep quality score of ∼7 for all conditions; for details see Table 1). To overcome any sleep inertia that might have existed, exercise tests were performed at 17h00 (Figure 1).

### 5-m Shuttle Run Test

**FIGURE 2:**
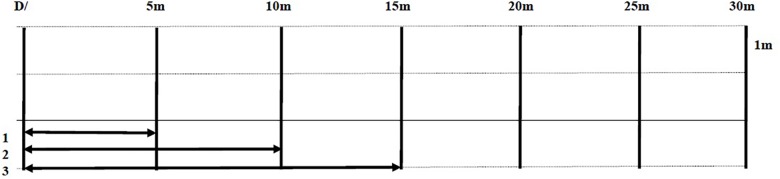
Schematic representation of the 5-m shuttle run test. D/, starting line.

At each session (N0, N25, N35, N45), participants performed a 5-min warm-up consisting of two min of easy running followed by three min of a series of dynamic exercises performed over a 10-m distance (i.e., internal–external hip rotations, lateral shuffles, high knees, heel kicks, lunges, and straight leg marches) at 17h00. Then, participants performed the 5-m shuttle run test. The test consisted of six repetitions of 30-s maximal shuttle sprints over 5 m, 10, 15, and 20 m alternatively (Figure 2) with a recovery period of 35 s in-between (Maso et al., 2002). The 5-m shuttle run test was utilized as it measures physical performance capacities that are related to speed and change of direction ability, and it stimulates both the aerobic and anaerobic pathways (Boddington et al., 2001; Maso et al., 2002).

For each turn, the participant must put a foot on the line without making a large arc (i.e., block to return using a 180° change of direction). The completed distance was recorded by an observer to the nearest 1 m. For the full description of the 5-m shuttle run test see Boddington et al. (2001).

From the distance data obtained in each test, the following parameters were calculated (Boddington et al., 2001):

- Total distance (TD) (m) = sum of distances covered during the 6 × 30 s shuttles
- Best distance (BD) (m) = highest distance covered during one of the 6 × 30 s shuttles
- Fatigue index (FI) (%) =

$$\text{FI }(\%)=\frac{\left[\frac{\text{shuttle }1+\text{shuttle }2}{2}-\frac{\text{shuttle }5+\text{shuttle }6}{2}\right]}{\frac{\text{shuttle }1+\text{shuttle }2}{2}}\times 100$$

### Rating of Perceived Exertion Scale (RPE)

The Borg CR10 scale ranges from 0 (very very light) to 10 (very very hard) and allows participants to give a subjective RPE for a physical task. The French version of the CR10 scale, validated by Haddad et al. (2013), was used in this study. Immediately after the completion of each 30-s repetition, the participant was shown the RPE scale and asked to say the number from 0 to 10 which best described their feeling of exertion at the completion of that 30-s effort.

The RPE value that was reported and used in the statistical analyses was the mean score during the 5 m shuttle run test and was calculated using the following formula:

$$\mathrm{RPE}=\frac{\sum \left(\mathrm{RPE after each}\;30\;\mathrm{s effort}\right)}{\mathrm{Number of repetitions}}$$

### Statistical Analyses

All statistical tests were performed using SPSS version 21.0 software (SPSS Inc., Armonk, NY). Mean and standard deviation (SD) values were calculated for each variable. G^∗^power software (Faul et al., 2007) was used to calculate the required sample size. Values for α were set at 0.05 and power at 0.80. Based on the study of Waterhouse et al. (2007) and discussions between the authors, effect size was estimated to be 0.7 (medium effect). The required sample size was fifteen.

The Shapiro–Wilk test revealed that sleep quality, RPE, FI, and BD data were normally distributed. Once the assumption of normality was confirmed, parametric tests were performed. Sleep quality, RPE, FI, and BD data were analyzed using a one-way analysis of variance (ANOVA) across four conditions (N0, N25, N35, and N45). When appropriate, *post hoc* coparisons were performed and the differences interpreted using a Bonferroni correction. The Shapiro–Wilk test was significant (*p* < 0.05) for TD; so a Friedman one-way ANOVA was used. Pairwise comparisons were conducted using a Wilcoxon test.

Effect sizes for the normally distributed variables, sleep quality, RPE, FI, and BD, were calculated as partial eta-squared (*η_p_*^2^) to estimate the meaningfulness of significant findings. To estimate practical relevance, partial eta-squared values of 0.01, 0.06, and 0.13 represent small, moderate, and large effect sizes, respectively (Clark-Carter, 1997). For TD, the effect size was estimated by the Kendall’s coefficient of concordance. Relationships between RPE and the three performance measures (BD, TD, and FI) were described using Bland and Altman (1995) correlations.

Significance was accepted for all analyses at the level of *p* < 0.05. Exact *p*-values have been given; results given as “0.000” in the statistics output have been reported as “<0.0005.”

Also, in order to calculate the percentage of gain or decrease for all parameters, a difference score or Δ score was calculated as follow:

$$\Delta \;(\%)=\left[\frac{\left(\mathrm{Higher value}-\mathrm{Minimal value}\right)}{\mathrm{Higher value}}\right]\times 100$$

## Results

### 5-m Shuttle Run Test

#### Best Distance (BD)

There was a significant main effect of Condition (*F* = 9.08, *p* < 0.0005, *η_p_*^2^ = 0.36 (large effect)) on BD. The *post hoc* analysis showed that BD was 6% higher (*p* = 0.03) in N25, and 9% higher (*p* < 0.0005) in N45 than in N0. However, BD in N35 was not different (*p* = 0.46) from BD in N0. In addition, BD in N45 was 6% higher (*p* = 0.01) than in N35 (Table 1).

#### Total Distance (TD)

A Friedman test conducted on TD data revealed a significant effect of Condition (test = 33.23, *p* < 0.0005, Kendall’s *W* = 0.65). Pairwise comparisons revealed that TD was 3% higher (*p* = 0.01) in N25, 3% higher (*p* = 0.009) in N35, and 8% higher (*p* < 0.000) in N45 than in the N0. In addition, TD was 5% higher (*p* = 0.001) in N45 than in N25, and 4% higher (*p* < 0.0005) than in N35 (Table 1).

#### Fatigue Index (FI)

There was no-significant main effect of Condition [*F* = 1.66, *p* = 0.18, *η_p_*^2^ = 0.09 (moderate effect)] for FI (Table 1).

### Rating of Perceived Exertion (RPE)

There was a significant main effect of Condition on RPE (*F* = 4.25, *p* = 0.009, *η_p_*^2^ = 0.20 (large effect)). The *post hoc* analysis showed that RPE were 19% lower (*p* = 0.04) in N45 than in N0; in addition, RPE were 20% lower (*p* = 0.01) in N45 than in N25 (Table 1).

### Sleep Quality During the Nap Opportunities

There was no significant main effect of Condition on sleep quality [*F* = 0.20, *p* = 0.81, *η_p_*^2^ = 0.01 (small effect)]. Mean values are in Table 1.

### Sleep Duration During the Night Preceding Testing

There was no significant main effect of condition on nighttime sleep duration preceding each experimental condition (*F* = 0.23, *p* = 0.87, *η_p_*^2^ = 0.01 (small effect)).

### Correlations Between RPE and Performance Measures (BD, TD, and FI)

The correlation between RPE and BD was not significant (*r* = -0.06; *p* = 0.623). However, there were significant correlations between RPE and TD (*r* = -0.43; *p* = 0.0015) and between RPE and FI (*r* = 0.50; *p* < 0.0005).

## Discussion

The main finding of the current study was that nap opportunity during the daytime has a positive effect on physical performance and perceived exertion in a high intensity shuttle run test that is performed after a normal night’s sleep (i.e., ≈7 h). Importantly, the beneficial effects seem positively related to the duration of the nap opportunity.

The benefits of napping on human performance previously have been confirmed following normal sleep (Hayashi and Hori, 1998) and partial sleep deprivation (Brooks and Lack, 2006) nights. These studies reported that naps improve cognitive (Brooks and Lack, 2006) and psychomotor performance (Verweij et al., 2016), enhance short-term memory and mood (Brooks and Lack, 2006) and reduce both subjective sleepiness and fatigue (Hayashi and Hori, 1998; Brooks and Lack, 2006).

A few studies have been conducted analyzing the impact of napping on athletic or physical performance(Waterhouse et al., 2007; Petit et al., 2014; Blanchfield et al., 2018; Hammouda et al., 2018; Keramidas et al., 2018; Suppiah et al., 2018; Daaloul et al., 2019). Waterhouse et al. (2007) showed a better sprint performance following a 30-min post-lunch nap in partially sleep-deprived athletes, with reduced mean time for both 2-m (−0.04 s) and 20 m (−0.09 s) sprints in comparison to a no-nap condition. However, Petit et al. (2014) reported that the a 20-min nap did not improve performance during the 30-s Wingate test after normal or 5-h phase advanced sleep. This contradiction might be explained because the nap duration was short or because the subjects were not sleep-deprived. In fact, the participants in the study of Waterhouse et al. (2007) slept only 4 h during the previous night with a 30-min napping opportunity prior to the exercise, while, in the study of Petit et al. (2014), the participants were not deprived of sleep and only 20 min of napping was allowed prior to the exercise. In addition, sleep inertia could affect the results of the previous studies. For example, the duration between the nap and the physical task was only 30 min in the study of Waterhouse et al. (2007). Sleep inertia usually takes approximately 1 h to dissipate (Brotherton et al., 2019). Therefore, in the present study, there are more than 2 h between the nap opportunity and the test session.

Hammouda et al. (2018) who demonstrated that an afternoon nap enhanced repeated-sprint performances after partial sleep deprivation, with the greatest beneficial effect using 90-min naps in comparison with 20-min naps. However, it has been shown that napping had no-effect on shooting performance, autonomic function (i.e., heart rate variability during simulated 20-min shooting competition), reaction time, and both 10 and 20-m sprint performance (Suppiah et al., 2018). Also, Suppiah et al. (2018) reported a significant negative effect of a nap (i.e., maximum duration 30 min) on 2-m sprint performance. These findings are in contradiction with the results of the present study and those of Waterhouse et al. (2007). These discrepancies could be explained by the reduced sleep at night in the study of Suppiah et al. (2018). In fact, the participants in the study of Suppiah et al. (2018) were high-level Asian youth athletes who trained regularly. Indeed, it is known that participation in vigorous exercise and sports training can elicit alterations of sleep patterns in adolescent and chronically shortened sleep durations (Suppiah et al., 2018), therefore, potentially carrying a greater sleep debt. Recently, Blanchfield et al. (2018) confirmed the present study’s observations, reporting that a short afternoon nap (34 ± 12 min in bed with 20 ± 10 min asleep time) improved endurance performance in runners who typically slept less than 7–9 h. Likewise, Keramidas et al. (2018) reported that a 30-min nap may attenuate the reductions in performance of an anaerobic endurance task. Recently, Daaloul et al. (2019) reported that a 30-min nap has a beneficial effect on performance during a karate-specific test and vertical jump tests after partial sleep deprivation.

The improvement of short-duration high-intensity performance after an afternoon short-nap could be explained by an improvement in alertness (Brooks and Lack, 2006) and a reduction of sleepiness (Waterhouse et al., 2007) and subjective fatigue (Brooks and Lack, 2006). In this context, Waterhouse et al. (2007) showed that the improvement in performance of both a 2-m test and a 20-m test is associated with an increase in alertness and a decrease in sleepiness following a 30-min nap. In the same way, Brooks and Lack (2006) reported that 10-min naps were beneficial for cognitive performance (i.e., letter cancellation test, symbol digit substitution task) and that this improvement is associated with an increase in vigor and a reduction in subjective fatigue. The present study reported that the RPE mean scores during the 5-m shuttle run test were significantly lower after N45 in comparison to N0 which could explain the increases in performance during this short-duration high-intensity exercise.

Concerning the best duration of nap opportunity during the daytime, the N45 seems to be the best duration to improve BD and TD and to reduce the RPE scores during the 5-m shuttle run test. In this context, TD increased by 3% after N25, 3% after N35 and 8% after N45. Likewise, BD increased by 9% following N45 vs. 6% after N25 and N35. Also, Δ RPE scores were better after N45 than after N25 and N35 (−19% vs. −6% and −1%, respectively). Thus, it could be posited that the participants performed better during N45 because they perceived the exercise to be less strenuous in that condition. The observed lower RPE scores for the longer nap opportunity could be explained by the amount of time spent in slow wave sleep. Indeed, slow-wave sleep is important for a good recovery and helps to restores physical damage and reduces stress and anxiety (Shapiro et al., 1981; Mulrine et al., 2012). However, as there was no-objective measurement of sleep, it is not possible to confirm whether this is actually the case in the present study.

Previous studies reported that improvements in physical performance were greater after naps longer than 30 min (Lovato and Lack, 2010; Hammouda et al., 2018). In this context, Hammouda et al. (2018) reported that the highest power, the lowest power, and the mean power during a running-based anaerobic sprint test were higher after a 90-min nap than after a 20-min nap. Also, Lumley et al. (1986) reported that, after one night of total sleep deprivation, naps of 60–120 min resulted in decreased sleepiness in healthy participants compared to a nap of 15 min.

It has been suggested that a long afternoon nap may be comparable to a night’s sleep in terms of sleep quality (Jiang et al., 2018). Also, it has been suggested that the improvement in physical performance after longer naps of 40–90 min could be explained by the greater amount of time spent in slow-wave sleep (Mulrine et al., 2012). Therefore, in the present study, the greater increases in performance during the 5-m shuttle run test in the N45 condition might be due to the role of slow-wave sleep.

The major limitation of the present study is the lack of an objective measurement of the previous night’s sleep and the sleep during the daytime nap opportunity (e.g., by the use of actigraphy or polysomnography). However, actigraphy is not effective to evaluate a short nap because it estimates sleep time by recording the movements of the body; in a short nap, it is possible that the participant does not move but is not sleeping. Thus, an objective measurement of the nap using polysomnography equipment would be required. However, polysomnography equipment may affect participants’ sleep for these short-period naps. Thus, a familiarization session to the nap using polysomnography equipment would be required to quantify the sleep quality during a short duration nap. Also, previous studies (e.g., Waterhouse et al., 2007) have used a questionnaire to quantify participants’ sleepiness (i.e., how they felt). These measures could help for better interpretations of the findings. Thus, the results of the present study must be confirmed by other studies while checking the states of sleepiness of the participants before performing a nap and utilizing some objective measurement of sleep quality (e.g., by the use of actigraphy or polysomnography).

## Strengths and Weaknesses

The present study is the first to investigate the effect of different durations of nap opportunity during the daytime on subsequent performance in individuals who had not experienced prior sleep disturbance. Above, we addressed the main limitation of the study, the lack of objective measurement if participants were able to fall asleep during each nap condition (as opposed to resting with eyes closed) as well as the lack of objective measures of sleep during the various nap opportunities. Since the scientific literature presents limited data about nap opportunity during the daytime and/or napping, the present study could help to improve the experimental design of future investigations.

## Conclusion

Results of the present study showed that a post-lunch nap opportunity has a beneficial effect on physical performance and perceived exertion. In addition, although N25 was similar to N45 for BD and N35 was similar to N45 for RPE, 45 min was the most effective afternoon nap opportunity duration (i.e., compared to 25 and 35 min) for improving performance and reducing RPE scores during the 5-m shuttle run test.

## Practical Recommendation

From a sports performance perspective, the implication of the present study is that athletes might benefit from a nap opportunity during the daytime before practice or before a competition, even if they have had a full night’s sleep.

## Ethics Statement

After receiving a description of the protocol, including potential risks, and benefits, participants gave their written consent to participate in the study. The criteria for participants’ inclusion were: they were non-smokers, they did not have pathological sleep disorders, they did not consume alcohol, and they were regularly engaged in training (e.g., jogging) for ≈1 h per day, 3 days per week. Additionally, to avoid any undesirable effect of sleep deprivation, participants were asked to keep usual sleep duration (≈7 h) throughout the experimental period. The present study was conducted according to the Code of Ethics for human experimentation, the Declaration of Helsinki (World Medical Association, 2013) and the protocol was fully approved by the Research Ethics Committee of the High Institute of Sport and Physical Education of Sfax, University of Sfax, Tunisia before the commencement of the assessments.

## Author Contributions

All authors listed have made a substantial, direct and intellectual contribution to the work, and approved it for publication.

## Conflict of Interest Statement

The authors declare that the research was conducted in the absence of any commercial or financial relationships that could be construed as a potential conflict of interest.

## Footnotes

1 http://www.randomization.com
